# The Cellular Behavior and SEM Evaluation of ProRoot and Root MTAs on Fibroblast L929

**Published:** 2006-10-01

**Authors:** Fariborz Moazami, Samira Shahsiah

**Affiliations:** 1 *Department of Endodontics, Dental School, Shiraz University of Medical Sciences, Shiraz, Iran*; 2 *Department of Endodontics, Dental School, Ahvaz University of Medical Sciences, Ahvaz, Iran*

**Keywords:** Cytotoxicity, Fibroblast, Mineral Trioxide Aggregate

## Abstract

**INTRODUCTION:** Mineral trioxide aggregate is being widely used for root end filling, pulp capping, perforation repair, and other endodontic procedures. Recently, a material similar to ProRoot mineral trioxide aggregate (PMTA) was developed in Iran named Root mineral trioxide aggregate (RMTA) with the claim of having the exact result of original MTA. The purpose of this study was to compare the amount of cell cytotoxicity of RMTA with PMTA at three different time periods using scanning electron microscopy (SEM) as well as the amount of cell viability at the above mentioned period.

**MATERIALS AND METHODS:** Three culture plates in each group were packed with a homogenous layer of PMTA and RMTA prepared according to manufacturers instruction. A plate of media without any material was used as control in each group. The material set for 72 h in CO2 incubator and 2x10 of fibroblast L929 was added to each plate. SEM evaluation with x800-3000 magnification and cell viability counting using trepan blue counting method were done after 48, 72, and 168 hours.

**RESULTS:** There was no significant difference between cell viability of PMTA and RMTA, although the amount of cells remained viable in PMTA group was higher at 48 and 168 hours while for RMTA, it was higher after 72 hours. The SEM evaluation showed that PMTA compared with RMTA has less porosity, but relatively similar amount of cell coverage was detected for both materials after 168 hours.

**CONCLUSION:** ProRoot and Root MTAs showed comparative biocompatibility while evaluated in vitro. The results suggest that RMTA can be used as an alternative for PMTA in clinical trials.

## INTRODUCTION

Root canal therapy in contemporary dentistry is considered as one of the most successful treatments. Different investigators have reported variety of success rates for root canal treatment which ranges up to 95% (1). Even though the success rate of non-surgical treatment is high, failures do occur. In recent years, there has been an increasing interest for the cases of failures in endodontic retreatment procedures; meanwhile, the success rate of this treatment protocol is varied from 62% to 98%which insist on the indications for surgical interventions (2). Periradicular surgery is the treatment of choice when non-surgical treatment is failed or existing restorative or prosthetic treatment is to be endangered by orthograde retreatment. Endodontic surgery encompasses surgical procedures performed to remove the causative agents for periradicular pathosis and to restore the periodontium to the biological and functional health status. One of the most arguments in periradicular surgery is about retrofilling material. In recent years, MTA has been identified as the material of choice amongst all others such as IRM, super EBA, cavit, etc due to its good properties (2). Many investigators indicated excellent properties such as the low cytotoxicity, biocompatibility, radiopacity, being hydrophilic, and its most interesting phenomenon which is deposition of new cementum and formation of hard tissue over the MTA surface, which is beneficial for healing of periradicular tissues (2-4). Recently, a material similar to PMTA was developed by Lotfi at Tabriz named Root MTA which was claimed to have the exact properties of original MTA (5). Different studies investigated the tissue response to its subcutaneous implantation (6-9) and its ability to prevent leakage, during a time period from 48h to 1 month (5,10) and all showed an acceptable results for RMTA in comparison with PMTA, but there has been no cell culture study searching the amount of it's cytotoxicity and its potency in creating a good environment for cells to be covered to its surface.

Therefore, the goal of this study was to investigate the amount of cytotoxicity of RMTA in comparison with PMTA at 3 different time intervals using SEM as well as the amount of cell viability at mentioned periods.

## MATERIALS AND METHODS

Fibroblast L929 was obtained from Tehran Pasteur Institute (C161) with 94% viability and 1.02/10 content. Cells were grown in DMEM (Dublecco`s Modified Eagle's Medium Nutrient Mixture F-12HAM) and 10% FBS (Fetal Bovine Serum). The cell vial was mixed with the medium plus 10% FBS and the cell suspension was gently transported to the 50 cm flask. The cells were passaged when a monolayer of cells was formed at the floor of the flask at 1 week which was the mean interval until the adequate consistency and density were obtained for further use. The cells were maintained at CO2 incubator with 95% air and 5% CO2 and the sixth collection of cells were used.

According to previous similar study (12) triple experiment had been done for each two experimental groups, which were as follows: 

Group 1: After sterilizing the PMTA powder (Dentsply, Tulsa, USA) in dry heat at 160^◦^C for about 1 hour (13), it was mixed with sterile distilled water according to manufacturer’s instruction (Powder to water weight ratio of 3 to 1). After mixing the powder and water on a sterile glass block and gaining a creamy consistency, the material was placed on the floor of prepared well (which was three for each group) by plastic instrument and was packed on the floor of each well with a wet sterile cotton pellet until a homogenous regular width of material was achieved. One well in each group was assumed as a control (media and cell without material); and overall, 4 wells had been placed in each group.

Group 2: RMTA (Salamifar Co., Tehran, Iran) was mixed according to manufacturer's instructions after being sterilized in the same way of group 1.Three wells in this group were packed following the procedures done in group 1 plus a well as a control.

All the wells in each group were incubated for 72 hours in a CO2 incubator with 95% air and 5% CO2. After this period, cell contained medium were added to each well on the content of 2x10 cell/ml (the cell content were the same as previous study) and after 48, 72 and 168 hours, two kinds of evaluation were done including SEM evaluation of each well under magnification of x800-3000 and cell viability counting, using Trepan blue staining.

The data were statistically analyzed by multifactorial variance and Mann-Whitney test.

## RESULTS

A confluent cell consistency of 2.5 × 10 cells/ ml was remained for the whole time of experiment in control group (7 days).

The results of viability counting for each time period are shown in table 1.

In the first interval the mean amount of cells remained viable for PMTA was 81.6, and 68.6 for RMTA. The results of this time period show a higher cytotoxic effect of RMTA compared with PMTA. This difference was not statistically significant (Table 1).

**Table1:** The mean of cell viability for each group at 3 different time periods

**Period** **Group**	**48 hours**	**72 hours**	**168 hours**
ProRoot MTA	81.6667 ± 5.85947	80.6667 ± 8.5040	56.2333 ± 48.1847
Root MTA	68.6667 ± 5.03322	84.0000 ± 3.6055	46.6667 ± 32.5166
Control	55.3333 ± 14.1892	53.0000 ± 41.1667	34.3333 ± 6.02771

**Figure 1:**
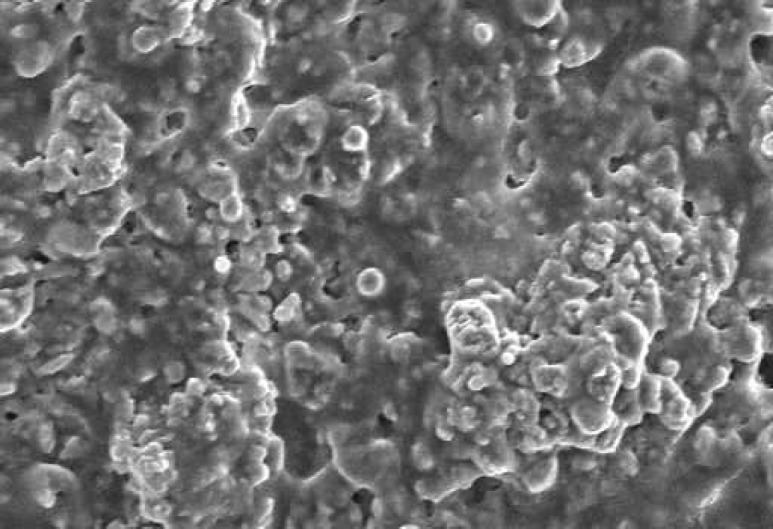
SEM of PMTA at 48 hours time period. The material has low porosity and the cells are covered homogenously on its all surfaces (×1000).

**Figure 2:**
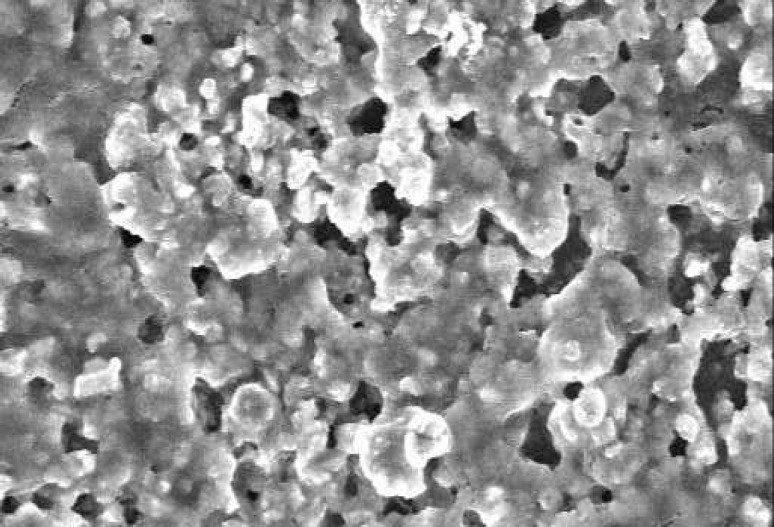
SEM of RMTA at 48 hours time period. The material has some porosity and the cells are covered the surfaces of material (×1000).

The second count interval revealed higher amounts of cell viability for RMTA (mean=84) versus PMTA (mean=80.6). Samples had been grossly enhanced compared with the first counting, indicating that the toxic effect diminished with time passes, however statistical analysis showed no significant difference between 2 experimental groups at this time period as well.

The amount of cells for RMTA group in third interval was lower compared with PMTA (40.6 versus 73.3). The results of this time period show high decrease in cell viability for RMTA while no prominent decrease was achieved in PMTA group compared with two other time periods. The results of this time period did not show significant difference as well.

**Figure 3:**
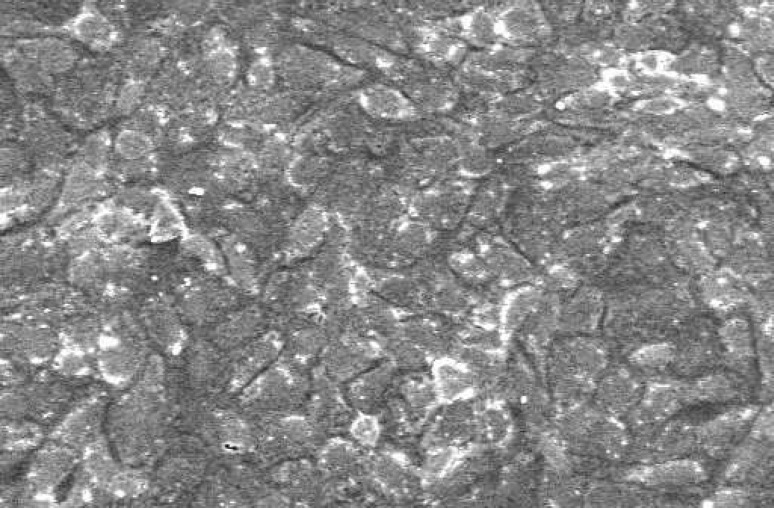
SEM of cells without material that served as control; Note the rhomboidal morphology of cells represents fibroblast. The cells are adhered on the floor of the plate (×1000).

**Figure 4:**
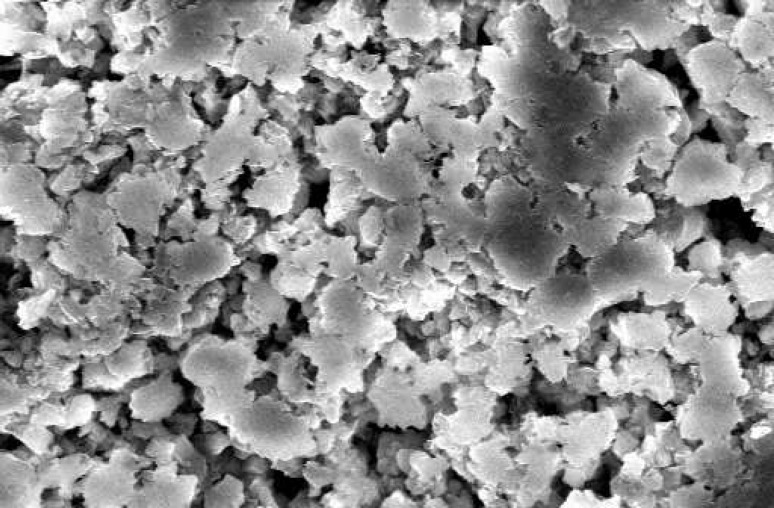
SEM of ProRoot MTA without cells. The surface of material is dense with no uneven pattern representing lack of cellular adhesion (×1000).

The SEM images at 48 hour time period are shown on Figure 1 and Figure 2. As the Figures show, the PMTA has less porosity and the cells are covered its all surfaces homogenously. The cells covered on RMTA have lower homogenicity and the cellular coverage is not so complete. Figure 3 presents cells without material which served as control. As the figure shows the cells are adhered on the whole surface of plate with the morphology of fibroblast. Figure 4 is the material without cells, the material has some porosity and there is no uneven surface indicating cellular adhesion.

**Figure 5:**
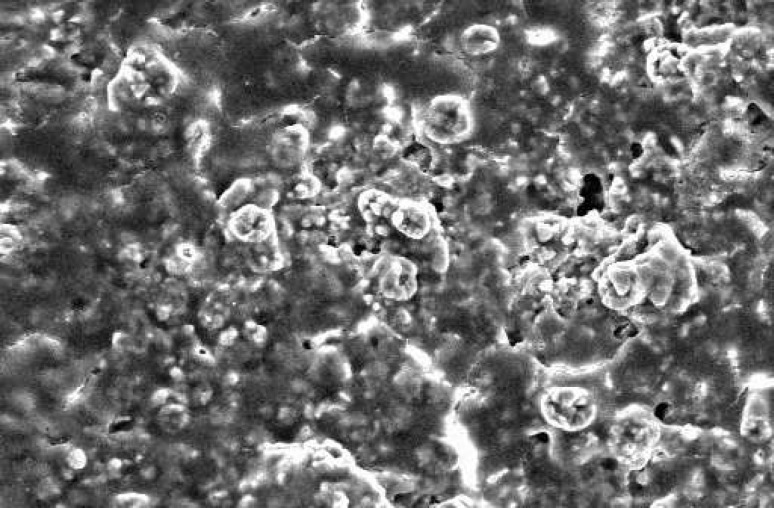
SEM of PMTA at 72 hours time period. The localization of cells over the surface of material is obvious and the pattern of cellular adhesion on the surface of material is globally with low porosity (×1000).

**Figure 6:**
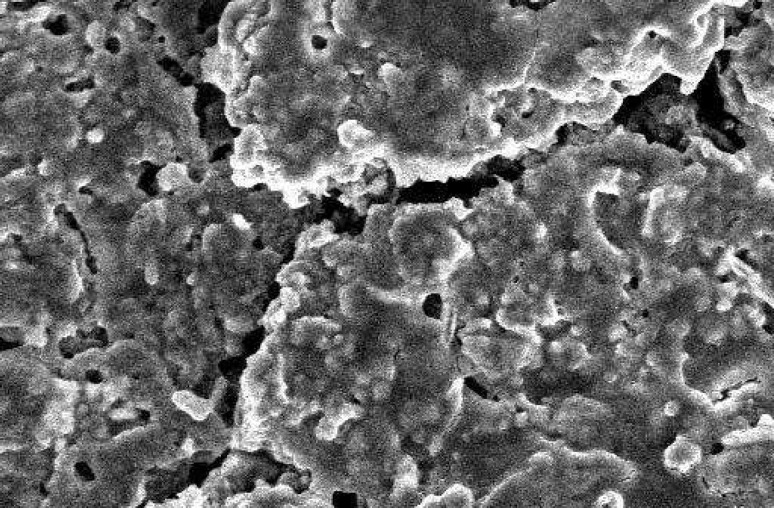
SEM of Root MTA at 72 hours time period; Note the porosity and the amount of cell coverage (×1000).

The amount of cellular coverage of PMTA after 72h is shown in Figure 5. The localization of cells over the surface of material is obvious and the porosity of it is much less than RMTA that has been shown on Figure 6. The surface of material is uneven with lots of porosity and the cells are adhered to its surface on a pattern not so similar to PMTA.

**Figure 7:**
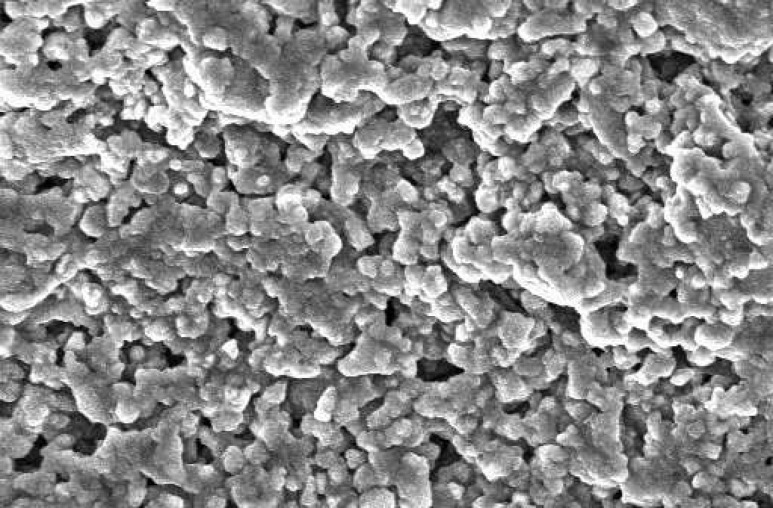
SEM of PMTA after 168 hours time period. The surface of material is so homogenous and the cells are adhered to the whole surfaces and formed nearly a confluent layer, indicating quiet complete adherence (×1000).

**Figure 8:**
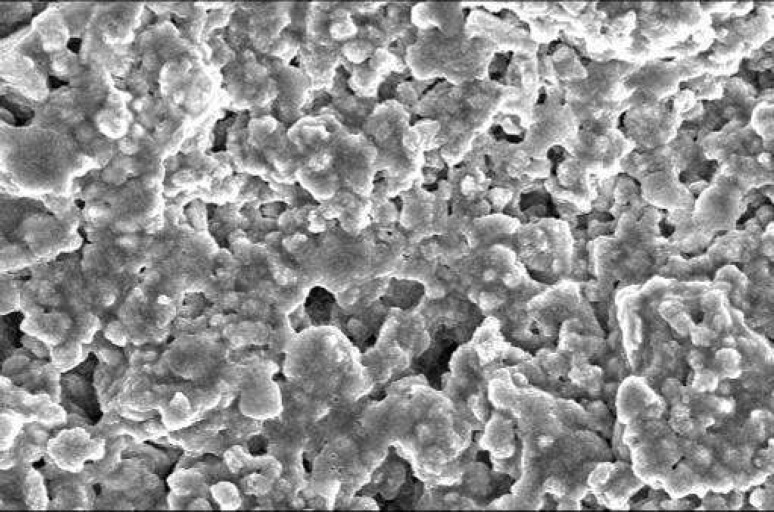
SEM of RMTA after 168 hours time period. The cells over the surface of Root MTA have an acceptable pattern compared with ProRoot MTA but they are not that even and the amount of cells adhered to the surface is quiet less compared with ProR oot MTA. Note that the material has some porosity on its surface.

Figure 7 shows the amount of cellular adhesion to PMTA after 1 week. The surface of material is so even and the cells are adhered to the whole surface and form nearly a confluent layer, indicating quiet complete adherence. The cells over RMTA are not that even and the amount of cells adhered to the surface is quite less compared with PMTA (Figure 8).

## DISCUSSION

This study was designed to compare two Root end filling materials currently used in periapical surgery. Various root end filling materials have been introduced, however, mineral trioxide aggregate _due to its excellent properties especially deposition of new cementum and formation of hard tissue on its surface_ is the most promising choice among others (2-4). Recently, a material similar to PMTA was developed by Dr. Lotfi in Tabriz named Root MTA which was claimed to have the same properties to original MTA (5). Different studies investigated the tissue response (6-9) as well as sealing ability of this new type of MTA (5,10,11). They showed acceptable results for RMTA, but there is no cell culture study searching the amount of its cell cytotoxicity and its potency in creating a good environment for cell cultures to get viability and to be covered over its surface. So PMTA which is the index of evaluation due to its approved result and RMTA were chosen in this study to evaluate their cytotoxicity at 3 different time periods (48, 72 and 168h). Among different methods for evaluating the toxic effect of endodontic materials such as subcutaneous implantation, cell culture and in vivo tissue tolerance reactions (12), cell culturing assay was chosen, because of the goal of this study which was the evaluation of cellular behavior and amount of cell toxicity of these materials.

There are different types of cell viability counting. MTT test and trypan blue are the most popular methods. The MTT Assay focuses on the capacity of mitochondrial dehydrogenizing enzymes in living cells to convert the yellow water-soluble tetrazolium salt 3,4-2,5 diphenyltetrazolium bromide (MTT sigma) into dark blue formazen crystals. The water insoluble product is stored in the cytoplasm of living test cells. The amount of formazen formed is directly related to the mitochondrial enzyme activity in a given cell line. After an appropriate incubation period of cells in the presence of test extracts, 20 microlitre of MTT solution would be added to each experimental well and the product would follows until the measurement of enzyme activity (13).

The trepan blue method is on the base of cell coloring pattern which is the index of differentiation between viable and dead cells. The method of counting these differentiated cells would be performed on Neobuere plate, measuring the mean amount of viable and dead cells. Trepan blue test was chosen for counting because of its simple and available procedure.

The results of this study showed no significant difference between these two materials at 3 different time periods; however there were different amount of cell viability for each time period.

The result of first counting at 48h showed higher amount of cell viability for PMTA than RMTA which is in agreement with other similar comparing studies of PMTA with other root-end filling materials such as Portland cement and MTA Angelus (14-15) but there is no literature about comparing the cell viability of RMTA and PMTA.

The SEM investigation of PMTA surface showed a cellular coverage with less porosity and its gradual completion to form a complete homogenous cover until the end of time period (168h). The result of RMTA was not similar to PMTA because of high material porosity and an uneven pattern of cellular coverage. These results are in agreement with other similar studies (16-19) reported the same pattern for PMTA.

After second counting, the mean amount of cell viability in this study was higher for Root MTA. However, there was no significant difference. These results are in coherence with the results of Jahromi and Shahi who reported better biocompatibility for RMTA compared with PMTA and amalgam (8). Other investigations on biocompatibility of reported no significant difference between RMTA and PMTA (6-7). It might be said that when time passes, the amount of cell cytotoxicity of material reduce. As the result of the second count shows, there was an increase in the amount of cell viability indicating less cellular toxicity of these materials when compared with 48h time period. The SEM evaluations coincide with what mentioned on first evaluation section features.

The 7 days counting showed higher amounts of cells remained viable for PMTA, although there was no significant difference. This might be attributed to gradual decrease in cytotoxicity of material with time passes.

## CONCLUSION

Our study showed no different cell cytotoxicity and cell viability for RMTA compared with PMTA and it might be a good alternative for PMTA in clinical trials.
